# Differential Effects of Controllable Stress Exposure on Subsequent Extinction Learning in Adult Rats

**DOI:** 10.3389/fnbeh.2015.00366

**Published:** 2016-01-12

**Authors:** Osnat Hadad-Ophir, Noa Brande-Eilat, Gal Richter-Levin

**Affiliations:** ^1^“Sagol” Department of Neurobiology, University of HaifaHaifa, Israel; ^2^The Institute for the Study of Affective Neuroscience (ISAN), University of HaifaHaifa, Israel; ^3^Department of Psychology, University of HaifaHaifa, Israel

**Keywords:** stress controllability, cued fear conditioning, contextual fear conditioning, extinction, resilience, infralimbic, interneuron, neuropeptides

## Abstract

Deficits in fear extinction are thought to be related to various anxiety disorders. While failure to extinguish conditioned fear may result in pathological anxiety levels, the ability to quickly and efficiently attenuate learned fear through extinction processes can be extremely beneficial for the individual. One of the factors that may affect the efficiency of the extinction process is prior experience of stressful situations. In the current study, we examined whether exposure to controllable stress, which is suggested to induce stress resilience, can affect subsequent fear extinction. Here, following prolonged two-way shuttle (TWS) avoidance training and a validation of acquired stress controllability, adult rats underwent either cued or contextual fear-conditioning (FC), followed by an extinction session. We further evaluated long lasting alterations of GABAergic targets in the medial pre-frontal cortex (mPFC), as these were implicated in FC and extinction and stress controllability. In cued, but not in contextual fear extinction, within-session extinction was enhanced following controllable stress compared to a control group. Interestingly, impaired extinction recall was detected in both extinction types following the stress procedure. Additionally, stress controllability-dependent alterations in GABAergic markers expression in infralimbic (IL), but not prelimbic (PL) cortex, were detected. These alterations are proposed to be related to the within-session effect, but not the recall impairment. The results emphasize the contribution of prior experience on coping with subsequent stressful experiences. Moreover, the results emphasize that exposure to controllable stress does not generally facilitate future stress coping as previously claimed, but its effects are dependent on specific features of the events taking place.

## Introduction

Fear conditioning (FC) and extinction are extensively studied in the context of stress related behaviors, and specifically in anxiety disorders. Impaired fear extinction is perceived as a central symptom of disorders caused by emotional trauma (Graham and Milad, 2014). Fear extinction is an expression of an active learning process (reviewed by Myers and Davis, 2002), in which a new, safe association is formed. The new “CS-no shock” association competes with the original acquired association but does not erase it (Bouton, 2002; Eisenberg et al., 2003). It was previously demonstrated that the extinction level can be affected by different factors. For example, it is attenuated by cocaine treatment (Burke et al., 2006) and sleep deprivation (Silvestri, 2005). Moreover, exposure to a stressor extrinsic to the context of FC was found to impair the extinction of fear (reviewed by Akirav and Maroun, 2007). Interestingly, it was demonstrated that exposure to escapable tail-shock results in facilitated extinction while inescapable shock damages it (Baratta et al., 2007). Understanding the factors that affect extinction acquisition is important not only because extinction of aversive memories is implicated in anxiety disorders and vulnerability to extreme stress (e.g., Lissek et al., 2005; Rauch et al., 2006; Hofmann, 2008), but also because of its involvement in the resilience to them. Stress resilience is more likely to be developed in individuals who display facilitated extinction (Haglund et al., 2007).

The actual, or apparent, control over a stressor is defined as the ability to alter the onset, duration, intensity or pattern of an aversive experience (Overmier and Seligman, 1967). The degree of behavioral control an organism exerts over a stressor critically determines the behavioral consequences of the stressful experience (Maier and Watkins, 2005). Various physiological alterations are evident under different controllability levels. For example, exposure to an uncontrollable stressor led to increased secretion of corticosterone (CORT; Weiss, 1971; Prince and Anisman, 1990; Akirav et al., 2001; Ilin and Richter-Levin, 2009) and increased the severity of gastric lesions (Weiss, 1968). Distinctively, the sense of control over a stressor has the ability to protect from the deleterious behavioral effects of stress and thus to potentiate a trait of stress resilience (Amat et al., 2006; Ilin and Richter-Levin, 2009; Lucas et al., 2014).

Stress controllability was found to induce alterations in several related brain regions. Elevation in noradrenaline release in the amygdala and hypothalamus was observed a few days after uncontrollable training, in comparison to rats exposed to controllable stress (Tanaka, 1999). In addition, 6 days training of two-way shuttle (TWS) avoidance task lead to pERK activation of the basolateral amygdala (BLA) after uncontrollable but not controllable stress (Ilin and Richter-Levin, 2009). Furthermore, controllability-dependent alterations in medial pre-frontal cortex (mPFC) and dorsal raphe nucleus were also reported (Amat et al., 2005; Rozeske et al., 2011). It was demonstrated that acquisition of stress controllability involved structural changes in the mPFC, as its outputs to the dorsal raphe nucleus, a stress-responsive brainstem nucleus, were enhanced (Maier and Watkins, 2005). It was proposed by Amat et al. (2005), that this output activation is responsible for behavioral changes and protective effects of behavioral control on stress-induced brainstem activity. Furthermore, an initial experience of controllable stress appears to alter the mPFC in such way that a subsequent uncontrollable stressor, which normally does not activate mPFC output, will now do so. Hence, gaining controllability in prior experience will result in a protective effect against the neurochemical and behavioral impacts of an uncontrollable stressor (Amat et al., 2006).

Notably, there exists a profound overlap in neuro-circuitry underlying both learning types, controllability over stress and extinction learning. Both contextual and cued FC and extinction were also repeatedly shown to involve the BLA, hippocampus and the mPFC (Maren, 2001; Milad et al., 2007; Maren et al., 2013). Moreover, the inhibitory effect of infralimbic (IL) projections to the central amygdala through the intercalated cells (ITC; Vertes, 2006) is crucial for extinction acquisition (McDonald et al., 1996; Smith et al., 2000; Berretta et al., 2005; Sierra-Mercado et al., 2011).

Interestingly, Izquierdo et al. (2006) have demonstrated that brief uncontrollable stress causes morphological alterations specifically in the IL, and not prelimbic (PL) cortex of the mPFC, and attenuated the cued fear extinction rate relative to non-stressed controls. This raises the possibility that prior experience of stress controllability may result in alteration of IL output to the amygdala, which could potentially facilitate the acquisition of FC extinction. The impact on IL output can rise from alteration in its excitation-inhibition balance, through changes in GABAergic interneuron transmission. The latter is known to be related to stress and anxiety states in the relevant circuitry (Kim et al., 2005; Bergado-Acosta et al., 2008; Jacobson-Pick et al., 2008; Yarom et al., 2008; Jacobson-Pick and Richter-Levin, 2010; Heldt et al., 2012). Specifically, GABAergic marker alterations also appear in the BLA after acute exposure to controllable and uncontrollable stress. A decreased expression of specific targets of interest (i.e., glutamate decarboxylase, *GAD65, GAD67)* was detected under controllable conditions, while uncontrollable conditions led to elevation in those genes (Hadad-Ophir et al., 2014). In addition, null mutation of *GAD65* in mice resulted in increased anxiety and resistance to conditioned fear extinction along with hyper-activation of the amygdala and the hippocampus (Stork et al., 2000, 2003; Bergado-Acosta et al., 2008; Müller et al., 2014, 2015). Interneurons also use neuropeptides, such as cholecystokinin *(CCK)* and neuropeptide Y *(NPY)*, as co-transmitters that exert profound effects on fear, anxiety, learned helplessness behavior and stress response (Ishida et al., 2007; Sherrin et al., 2009; Lach and de Lima, 2013; Serova et al., 2014), and stress was found to affect their expression as well (Hadad-Ophir et al., 2014).

We previously developed a behavioral task based on prolong exposure to the TWS avoidance task, which resulted in gained controllability (Ilin and Richter-Levin, 2009; Lucas et al., 2014). In the current study, we employed this model to assess the long-term impact of prolonged controllable stress exposure on subsequent fear extinction. We first verified the behavioral differences between the controllable stress group and a control group. Next, cued or contextual FC was conducted, followed by an extinction training in order to further examine if the beneficial effects of prolonged controllable training will be also evident in fear extinction acquisition. In addition, in order to assess stress controllability-induced alterations preceding the FC and extinction training, we evaluated messenger RNA (mRNA) expression levels of selected GABA transmission related targets in the mPFC, 2 weeks after completion of the controllable training.

## Materials and Methods

### Animals

Male Sprague–Dawley rats were obtained at an age of 60 PND (weight 275–300 g) from Harlan Laboratory (Jerusalem, Israel). Animals were maintained in groups of 4 on a 12 h light: 12 h dark cycle (lights on 07.00 am) with food and water *ad libitum*. After 5 days of acclimation rats were assigned to behavioral training. All experiments were carried out during the light phase (9.00 am–5.00 pm), in accordance with the NIH guidelines for the care and use of laboratory animals and were approved by the University of Haifa ethical committee (Ethical No. 230/11).

### Behavioral Protocol

#### Experimental Design

**Figure 1:**
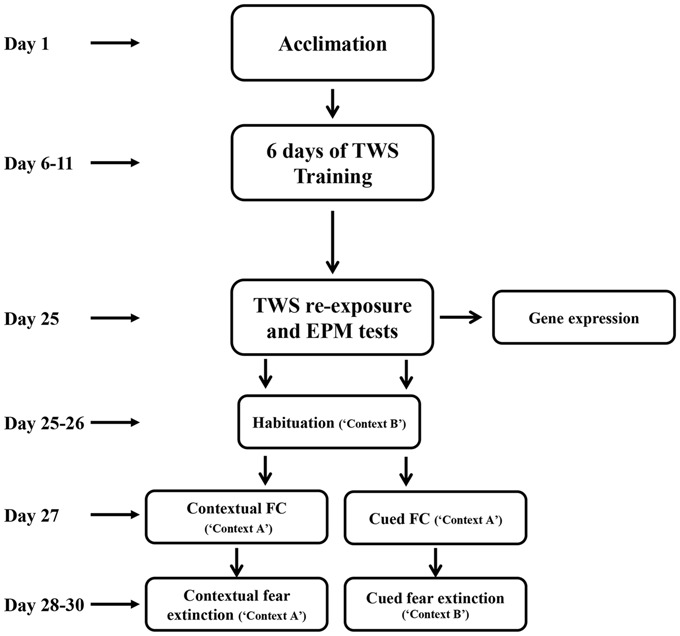
**Experimental design.** After 5 days of acclimation, rats went through active avoidance training in a two-way shuttle (TWS) avoidance task. Two weeks after the end of TWS training animals’ behavior was assessed by TWS re-exposure and elevated plus maze (EPM) tests. Two days later, animals underwent cued or contextual fear-conditioning (FC) and extinction training.

After acclimation, rats were randomly assigned to two different groups. “Controllable” group (*n* = 18) went through active avoidance training in a TWS avoidance task. “Unexposed” group (*n* = 27) were placed in the TWS box for an equivalent amount of time of free exploration as the controllable group. This group was not exposed to any tones or shocks during the training period. Two weeks after the end of TWS training animals’ behavior were assesses by TWS re-exposure and elevated plus maze (EPM) tests. Two days after, animals went through cued or contextual FC and extinction training (Figure 1).

#### TWS Apparatus

The TWS avoidance box was a rectangular chamber (60 × 26 × 28 cm), divided by an opaque partition with a passage (10 × 8 cm) into two equal size compartments, within a dimly-lit, ventilated, sound-attenuated cupboard (Panlab, Harvard Apparatus, Barcelona, Spain).

#### TWS Training

TWS avoidance training (adapted from Tsoory and Richter-Levin, 2006) was composed of 6 days with 50 trials per day. Rats were given 10 min of free exploration period in the first day and 1 min of exploration in the next 5 days. Shuttling number between the chambers served as a measure for exploration level. After exploration period in each training day, training session started with the delivery of the conditioned stimulus (CS; 3000 Hz tone, 75 db, 10 s), immediately followed by the unconditioned stimulus (US; electrical foot-shock, 0.8 mA, 10 s maximum) with an inter trial interval (ITI) of 30 s Responses of the rats during each trial were divided into three types: avoidance (shuttling during the tone and thus avoid the shock), escape (Esc; shuttling during the shock), and Esc failure (animals do not perform shuttling either during the tone or shock). Rats’ location was tracked automatically via the weight-sensitive metal grid floors in both compartments and was collected for offline-analysis via the ShuttAvoid Software (Panlab, Harvard Apparatus, Barcelona, Spain). The criterion for successful avoidance learning was set as reaching an avoidance rate of more them 50% during the training. Rats that haven’t reached the criterion were excluded from the analysis.

#### TWS Re-Exposure Test

Two weeks after completing TWS training, rats’ behavior was assessed in the TWS box. After 3 min of free exploration in the TWS, rats were presented with 10 presentations of the CS (3000 Hz tone, 10 s maximum) separated by an ITI of 30 s.

#### EPM

Immediately after the end of TWS re-exposure test, all rats were tested in the EPM, a cross-shaped maze 70 cm above the floor, consisting of two opposing open arms and two opposing closed arms (with 30 cm high walls and no roof; total length of arms 112 cm, 8 cm wide). Following 5 min of habituation to the room in a standard cage, each animal was placed in the center of the maze, facing an open arm. Animal was allowed to explore the arena freely for 5 min while its behavior was recorded via the Etho-Vision video tracking system (Noldus Information Technology, Wageningen, Netherlands). Time spent, distance traveled and frequent of entries in the closed and open arms were collected, analyzed, and served as measures of anxiety-related behavior.

#### FC and Extinction Training

After 2 days in the home cage animals went through either cued (unexposed, *n* = 9; controllable, *n* = 6) or contextual (unexposed, *n* = 8; controllable, *n* = 6) FC and extinction. FC boxes consisted of a square chamber (24 × 26 × 27 cm. Panlab, Harvard Apparatus, Barcelona, Spain). Both FC protocols were conducted in “context A” (grid-floor, black walls and full lighted chamber), rats were placed in the FC box and were allowed to explore for 120 s Then, rats were exposed to three CS (10 slight), followed immediately by an US (1 s 0.6 mA shock). It is important to note that we used light instead of tone as a CS in the cued FC and extinction, in order to avoid generalization with respect to the TWS training tone (reviewed by Myers and Davis, 2007). Rats that went through contextual FC were put in “context A” for equivalent amount of time as in cue FC, and received three shocks, separated by equal ITIs as in the cued FC protocol.

In the following 3 days rats were subjected to an extinction protocol. During cued fear extinction protocol 10 CSs were presented (every two CSs were later averaged and referred as “Blocks”), separated by a 120 s interval in “context B” (white walls surrounded by a round transparent Plexiglas, metal plain served as the floor, Plexiglas door and dim light. Walls and floor were cleaned with 30% ethanol). Extinction of contextual FC took place in “context A”, in which the rats were put for an equivalent amount of time as in the cued extinction, with no cue presentation.

Freezing levels during FC and extinction were measured automatically via the weight-sensitive floor and were collected for offline-analysis via the Freezing Software (Panlab, Harvard Apparatus, Barcelona, Spain). Analysis of FC and extinction evaluated freezing levels during CSs presentation in the cued paradigm and at equivalent time periods at the contextual paradigm. Two days prior to FC procedure, rats were habituated for 10 min to “context B”.

### Brain Preparation

Six hours after TWS and EPM behavioral tests, a subset of animals (unexposed, *n* = 10; controllable: *n* = 6) were decapitated, their brains were removed and immediately snap-frozen on powdered dry ice and stored at –80°C. Brains were mounted on the cerebellum in the cryostat apparatus (chamber temperature −20°C). The brain was sliced until the mPFC was reached (3.2 mm from Bregma; Paxinos and Watson, 1998). With stainless steel puncher tissue punches of IL and PL sub regions were taken for molecular analysis of alterations in GABA-related gene expression.

### RNA Isolation and Quantitative Real-Time PCR

Sample lysis and subsequent isolation of total RNA via a spin column system was conducted with the RNA Purification Kit, (NORGEN, Thorold, ON, Canada) according to manufacturer’s instructions, including steps for removal of genomic DNA. RNA samples were stored at −80°C until further processing. First-strand synthesis of cDNA was performed with the OuantiTech Reverse Transcription kit (QIAGEN, Hilden, Germany), following an additional step for removal genomic DNA, in the presence of Ouantiscript RT buffer 5× as well as RT primer mix (oligo-dT and random primers) and Ouantiscript reverse transcriptase (Omniscript and Sensiscript Reverse Transcriptase with RNase inhibitor) at 42°C for 20 min, followed by enzyme inactivation at 95°C for 3 min. A 1:5 dilution of cDNA samples was used for determination of expression levels of selected target genes by quantitative PCR using the ABI Prism Step One real time PCR apparatus (Life Technologies, Darmstadt, Germany) and TaqMan^®^ reagents with predesigned assays for *GAD65* (Gad2; assay ID Rn00561244_m1), *GAD67* (Gad1; assay ID Rn00566593_m1), *NPY* (assay ID Rn00561681_m1), *CCK* (assay ID Rn00563215_m1) and the housekeeping gene glycerinaldehyd-3-phosphat-dehydrogenase (*GAPDH*; endogenous control, Life Technologies, Darmstadt, Germany). Target and housekeeping genes were labeled with different fluorescent dyes, allowing for quantitative multiplex PCR. Samples were run in triplicate assays, consisting of 50 cycles of 15 s at 95°C and 1 min at 60°C, preceded by a 2 min decontamination step at 50°C with Uracil-N-Glycosidase and initial denaturation at 95°C for 10 min.

For data analysis, the mean cycle threshold (CT) was determined for each triplicate assay and relative quantification (RQ) of each target gene was conducted with the ddCT method (Livak and Schmittgen, 2001), normalizing each sample to the overall content of cDNA using GAPDH as an internal control (dCT; dCT = (CT (target gene)) − (CT (GAPDH))). Normalization of all ddCT values was done relative to unexposed group with ddCT = dCT (sample) − mean dCT (unexposed group). Transformation to RQ values for a specific target gene and area was done according to RQ = 2^−ddCT^.

### Statistical Analysis

Paired or independent samples *t*-tests and one-way or mixed model repeated-measures ANOVA were conducted on normal distributed data sets (assessed by Shapiro-Wilk’s test). Degrees of freedom were corrected when necessary in *t*-test or repeated measures ANOVA, according to Leven’s test or Mauchly’s test, respectively. When found a significant interaction effect at mixed-model repeated measures ANOVA, follow up analysis was conducted using one-way repeated measures ANOVA when asking to asses trends in separate groups. Additionally, independent samples *t*-tests were conducted when asking to assess between-groups simple effects. Variables with distribution deviating from normality were tested using nonparametric tests (specifically, Mann-Whitney U test).

## Results

### Prolonged Exposure to Controllable Stress Within the TWS

**Figure 2:**
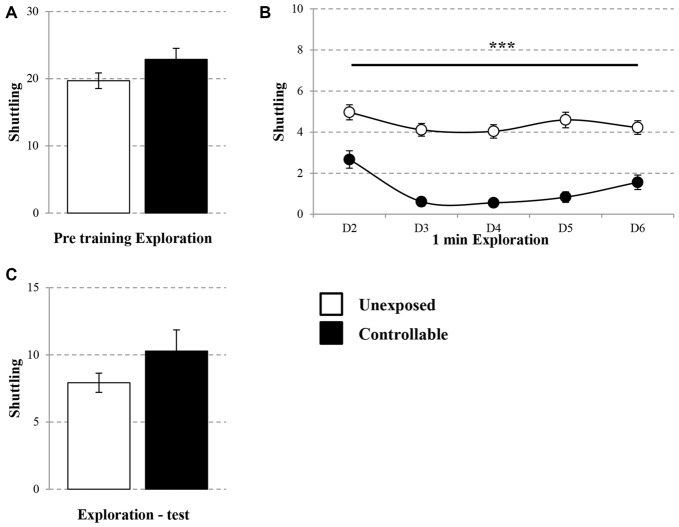
**Exploration rate in the TWS.** Exploration rate of controllable and unexposed groups were measured before and during exposure to TWS training and in TWS re-exposure test. **(A)** Exploration rates in the TWS were equal for both groups 10 min prior to training. **(B)** The first minute of exploration in the beginning of each day of training was decreased in the controllable group along training while unexposed animals maintained the same exploration rate. **(C)** Two weeks after the end of TWS training no difference in exploration rate was detected between groups. Values presented as mean ± SEM. ***significant difference between groups with *p* < 0.001.

On the first day, 10 min pre-training exploration in the TWS was equal in both groups. Controllable and unexposed animals shuttled freely in the TWS arena (Mann-Whitney U test; *U* = 194.00, *Z* = −0.41, n.s; Figure 2A). In addition, in the following training days a session × group effect was found for the exploration rate (Repeated measures test; *F*_(4,172)_ = 3.05, *p* < 0.05; Figure 2B), whereas exposure to TWS training significantly decreased shuttling during the first 1 min of exploration in each day (*t*-test; D2: *t*_(43)_ = 4.08, *p* < 0.001; D3: *t*_(43)_ = 9.30, *p* < 0.001; D4: *t*_(43)_ = 9.07, *p* < 0.001; D5: *t*_(43)_ = 8.20, *p* < 0.001; D6: *t*_(43)_ = 5.36, *p* < 0.001). When animals freely explored the TWS arena 2 weeks after the end of the TWS training, no difference was detected between groups (*t*-test; *t*_(43)_ = −1.36, n.s; Figure 2C).

**Figure 3:**
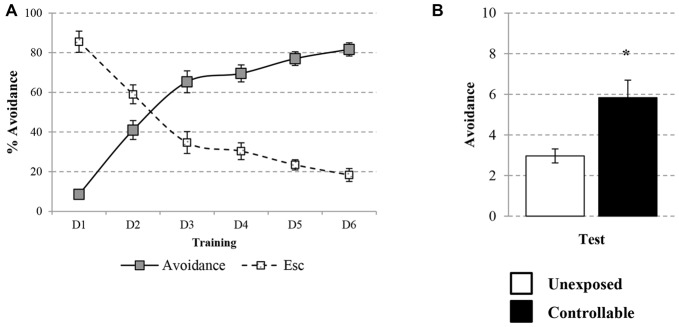
**Avoidance response in the TWS.** Avoidance and Esc responses were measured for the controllable group during training in the TWS and for both groups in the TWS re-exposure test. **(A)** Learning curve of controllable group during prolonged exposure to controllable conditions was improved along days while the rate of Esc response was decreased. **(B)** Two weeks after the end of TWS training controllable animals exhibited more avoidance responses in comparison to the unexposed group. Values presented as mean ± SEM. *significant difference between groups with *p* < 0.05.

**Figure 4:**
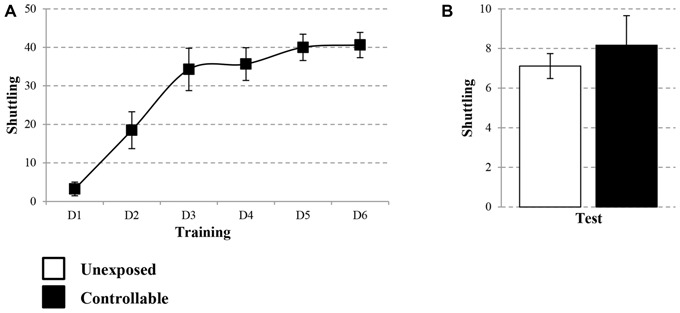
**Number of shuttles during ITIs within TWS exposure.** Number of shuttles during ITIs of controllable group was measured during exposure to TWS training and for both groups in TWS re-exposure test. **(A)** In the controllable group the number of shuttles during training increased with time. **(B)** Two weeks after the end of TWS training both groups exhibited similar shuttling rates during ITIs. Values presented as mean ± SEM.

The learning curve of the controllable group was improved during training in the TWS as successful avoidance responses gradually increased (One way repeated-measures ANOVA; *F*_(3,73)_ = 128.31, *p* < 0.001), while Esc responses decreased (One way repeated-measures ANOVA; *F*_(3,48)_ = 52.73, *p* < 0.001; Figure 3A). The acquired responses persisted: when examined 2 weeks later, controllable animals significantly exhibited higher levels of avoidance responses compared to unexposed animals (Mann-Whitney U test; *U* = 133.50, *Z* = −2.56, *p* < 0.05; Figure 3B). The number of shuttles during the ITIs was also increased during training (Repeated measures test for days in the TWS; *F*_(5,85)_ = 26.90, *p* < 0.001; Figure 4A) and 2 weeks later both groups exhibited similar shuttling rates during the ITIs (*t*-test; *t*_(43)_ = −0.65, n.s; Figure 4B).

### EPM Test

**Figure 5:**
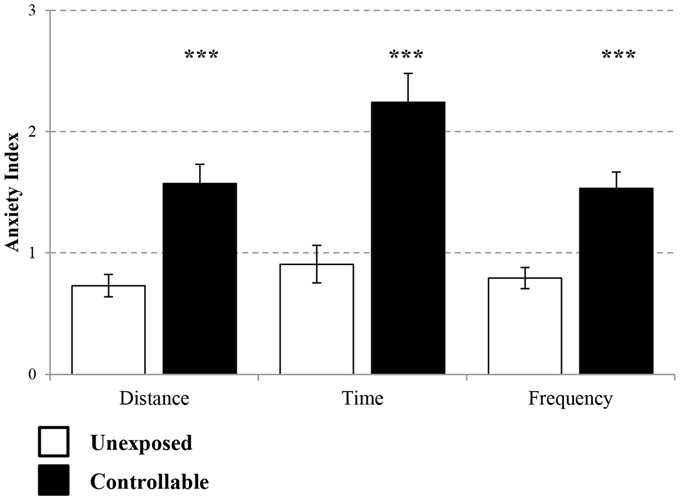
**Anxiety Index levels in the EPM.** Two weeks after the end of TWS training anxiety indices of distance covered, duration and frequency of entries to open vs. closed arms were measured. The controllable group displayed decreased anxiety levels in all parameters in comparison to unexposed animals. Values presented as mean ± SEM. ***significant difference between groups with *p* < 0.001.

The controllable group exhibited lower levels of anxiety as reflected by EPM behavioral test in parameters of distance covered (Mann-Whitney test; *U* = 71.00, *Z* = −3.99 *p* < 0.001), time spent (Mann-Whitney test; *U* = 67.00, *Z* = −4.08, *p* < 0.001) and frequency of entries (*t*-test; *t*_(43)_ = −4.80, *p* < 0.001) in open vs. closed arms in comparison to unexposed group (Figure 5).

### FC and Extinction

A significant block × group effect was observed for cued FC freezing levels (Mixed model repeated-measures ANOVA; *F*_(2,26)_ = 7.78, *p* < 0.01). Accordingly, follow up analysis was performed in order to examine the changes in freezing during the course of training in each of the groups separately. A significant increase during the cue presentations was found in both groups (One-way repeated measures ANOVA; unexposed: *F*_(1,9)_ = 370, *p* < 0.001, controllable: *F*_(2,10)_ = 18.39, *p* < 0.001). In order to examine differences between the groups in each block, *post hoc* analysis was performed. However, no significant difference was observed at any of the groups (*t*-tests; FC: Block 1: *t*_(5)_ = −2.39, n.s; Block 2: *t*_(6)_ = −1.60, n.s; Block 3: *t*_(5)_ = 1.32, n.s).

Taken together, and considering that both groups reached high percentage levels of freezing (100% with no standard deviation and 95.37 ± 8.57 for the unexposed and controllable groups, respectively), it can be concluded that both groups properly acquired FC learning to the same level.

A group × block interaction effect was evident in each of the extinction days (Mixed-model repeated measures ANOVA; Day 1: *F*_(2,32)_ = 13.71, *p* < 0.001; Day 2: *F*_(4,52)_ = 3.25, *p* < 0.05; Day 3: *F*_(5,52)_ = 4.13, *p* < 0.01). Thus, in each of the extinction days, there was a difference in the trend of freezing levels reduction between the groups. Follow-up analysis showed that indeed both groups displayed a reduction in within-session freezing levels at all days (One-way repeated measures ANOVA; Day 1: unexposed: *F*_(4,32)_ = 7.53, *p* < 0.001, controllable: *F*_(4,20)_ = 17.56, *p* < 0.001; Day 2: unexposed: *F*_(4,32)_ = 11.09, *p* < 0.001, controllable: *F*_(4,20)_ = 22.58, *p* < 0.001; Day 3: unexposed: *F*_(4,32)_ = 11.13, *p* < 0.001, controllable: *F*_(4,20)_ = 17.45, *p* < 0.001). *Post hoc* comparisons of between-groups differences in each block showed that the interaction effect stemmed from a steeper reduction rate in the controllable group compared to the unexposed group. While at the first block of every extinction day there was no difference between the groups (*t*-test; Day 1: *t*_(13)_ = −0.09, n.s; Day 2: *t*_(6.20)_ = 1.08, n.s; *t*_(13)_ = −0.49, n.s), towards the end of the session (at either the 4th, 5th blocks, or both) the freezing levels of controllable group were significantly lower compared to the unexposed group (*t*-test; Day 1, 4th block: *t*_(13)_ = 3.30, *p* < 0.01; 5th block: *t*_(13)_ = 8.77, *p* < 0.001; Day 2: 4th block: *t*_(13)_ = 3.92, *p* < 0.01; Day 3: 5th block: *t*_(13)_ = 2.76, *p* < 0.05). Hence, we can conclude that while both groups showed reduction in freezing levels, the controllable group displayed faster and greater within-session extinction level.

**Figure 6:**
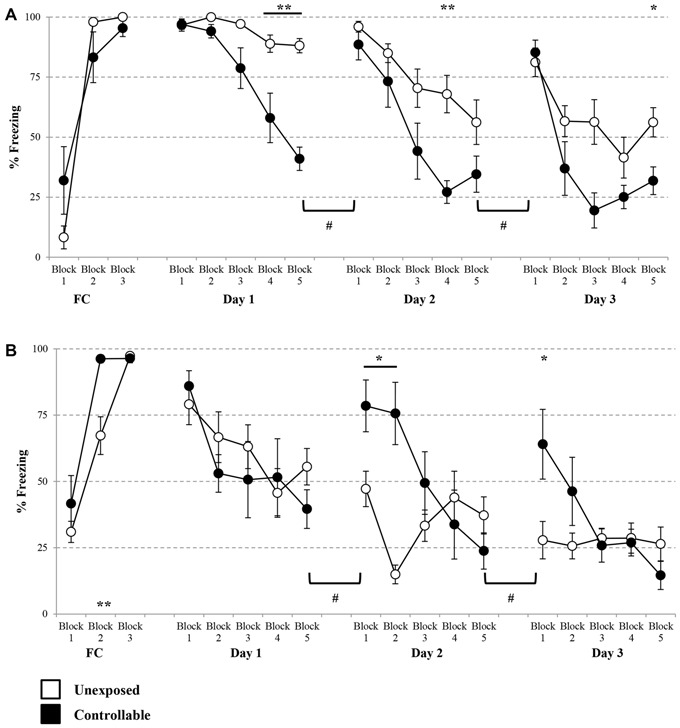
**Cued and contextual FC and Extinction.** Two days after TWS re-exposure and EPM tests, animals underwent cued or contextual FC and extinction. **(A)** Cued FC and extinction. Freezing levels during cue presentations were significantly increased in both groups during FC acquisition. In each of the extinction days, there was a difference in the trend of freezing levels reduction between the groups. Both groups displayed a reduction in within-session freezing levels at all days; however a steeper reduction rate was observed for the controllable group in comparison to the unexposed group. Poor recall, limited to the controllable group, was detected between days of extinction. **(B)** Contextual FC and extinction. Both groups successfully acquired extinction of contextual FC, with faster learning for the controllable group. In the first day of extinction, both groups showed a comparable reduction of within-session freezing levels. In the last 2 days of the extinction training, there was a difference in the trend of freezing level reduction between the groups. That difference resulted from a steeper reduction rate in the controllable group, compared with the unexposed group. At the first two blocks of each extinction day, freezing levels of the controllable group were elevated in comparison to the unexposed group. In addition, the controllable group displayed poor recall response and increased freezing levels between days of extinction, while freezing levels of the unexposed group did not change between days. Values presented as mean ± SEM. *significant difference between groups with *p* < 0.05, **p < 0.01; ^#^significant difference between measures of controllable group with *p* < 0.05.

In addition, when examining “recall” of extinction learning between days, a significant difference between second and first day, was revealed (Mixed-model repeated measures ANOVA; group × block interaction effect: *F*_(1,13)_ = 52.82, *p* < 0.001). Follow up analysis of the recall effect in each group separately, revealed that freezing levels did not change significantly between first and second day of extinction for the unexposed group (Related-samples sign test; *Z* = 1.06, n.s), while freezing levels of the controllable group were significantly elevated between days (Related-samples sign test; *Z* = 2.04, ^#^*p* < 0.05, ^#^significant difference between blocks for controllable group). When analyzing the recall effect between second and third day, there was not a significant group × block effect (Mixed-model repeated measures ANOVA; *F*_(1,13)_ = 4.09, *p* = 0.064), however, when examining the difference between the groups the same effect was found. The unexposed group showed no difference between second and third day while controllable group did (unexposed: n.s; controllable: ^#^*p* < 0.05, ^#^significant difference between blocks for controllable group; Figure 6A).

Analysis of contextual FC revealed that both groups reached high percentage levels of freezing by the end of the session (unexposed: 97.30 ± 3.74, controllable: 96.37 ± 4.02), however a significant block × group effect was found (Mixed model repeated-measures ANOVA; *F*_(2,24)_ = 4.44, *p* < 0.05). Follow-up analysis was performed in order to examine the change in freezing during the course of the training in each of the groups separately. A significant increase was found in both groups (One-way repeated measures ANOVA; unexposed: *F*_(2,14)_ = 57.48, *p* < 0.001, controllable: *F*_(1,5)_ = 29.05, *p* < 0.01). Taken together, we concluded that both groups properly acquired FC. In order to examine differences between the groups at each FC block, *post hoc* analysis was performed. A significant difference was observed only at the second block (*t*-tests; Block 1: *t*_(12)_ = −1.047, n.s; Block 2: *t*_(12)_ = −3.44, *p* < 0.01; Block 3: *t*_(12)_ = 0.45, n.s). Despite that and due to high levels of freezing at the end of FC training it is safe to assume that contextual FC was achieved. At the first extinction day, no significant group × block interaction effect was found (Mixed-model repeated measures ANOVA; *F*_(4,48)_ = 0.966, n.s). However, there was a significant main effect for the training block (Mixed-model repeated measures ANOVA; *F*_(4,48)_ = 6.06, *p* < 0.001). In addition, no significant group main effect was found (Mixed-model repeated measures ANOVA; *F*_(1,12)_ = 0.48, n.s). Thus, we can conclude that both groups showed reduction of freezing levels within the first extinction session to the same extent. Distinctively, at the second and 3 days of the extinction training, a significant group × block interaction effect was found (Mixed-model repeated measures ANOVA; Day 2: *F*_(4,48)_ = 12.71, *p* < 0.001; Day 3: *F*_(2,27)_ = 5.09, *p* < 0.05). Thus, in the last 2 days of the extinction training, there was a difference in the trend of freezing level reduction between the groups. Follow-up analysis showed that in the second extinction day both groups displayed a reduction in within-session freezing levels (One-way repeated measures ANOVA; unexposed: *F*_(4,28)_ = 4.65, *p* < 0.01, controllable: *F*_(4,20)_ = 15.81, *p* < 0.001). *Post hoc* comparisons of between-groups differences in each of the blocks showed that the interaction effect stemmed from a steeper reduction rate in the controllable group, compared with the unexposed group. While at the first two blocks of the extinction day, freezing levels of the controllable group were elevated compared to the unexposed group (*t*-test; Block 1: *t*_(12)_ = −2.75, *p* < 0.05; Block 2: *t*_(12)_ = −4.95, *p* < 0.01), at the subsequent blocks no significant differences were observed (*p* > 0.05 for blocks 3–5).

Follow-up analysis of the interaction effect at the third day of contextual fear extinction showed that the controllable group displayed a reduction in freezing levels, while the unexposed group did not (One-way repeated measures ANOVA; unexposed: *F* < 1, n.s, controllable: *F*_(4,20)_ = 5.77, *p* < 0.01). *Post hoc* comparisons of between-groups differences in each block showed that, similarly to the second day, at the first block of the extinction day freezing levels of the controllable group were elevated compared to the unexposed group (*t*-test; *t*_(12)_ = −2.60, *p* < 0.05), and at the subsequent blocks there was no significant difference (*p* > 0.05 for blocks 2–5).

In addition, when examining “recall” of extinction learning between days, mixed-model repeated measures ANOVA revealed a significant group × block interaction effect between the first and the second day (*F*_(1,12)_ = 8.25, *p* < 0.05), and also between the second and third day (*F*_(1,12)_ = 11.94, *p* < 0.01). Follow-up analysis of recall interaction effect in each group separately, revealed a dissociation between the groups. Between day 1 and day 2 and between day 2 and day 3, freezing levels of the unexposed group did not change significantly, while freezing levels of the controllable group increased (^#^*p* < 0.05, significant difference between blocks for controllable group; Figure 6B).

### Gene Expression in the mPFC

**Figure 7:**
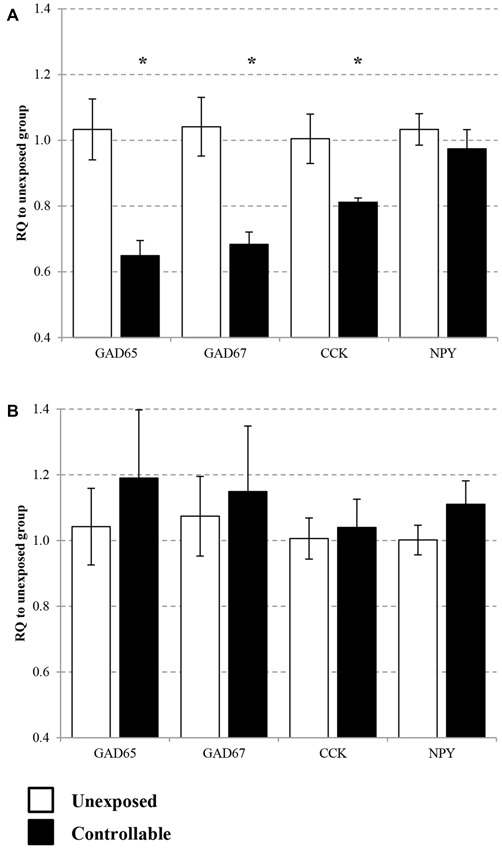
**Selective change of GABA-related gene expression in the medial pre-frontal cortex (mPFC).** Differential messenger RNA (mRNA) expression levels of the selected GABAergic marker genes were detected in distinct mPFC sub-regions 6 h after TWS re-exposure. **(A)** In the infralimbic (IL) *GAD65, GAD67* and cholecystokinin (*CCK*) mRNA levels were decreased 2 weeks after the end of controllable conditions training. No significant differences between the groups were observed in neuropeptide Y (*NPY*) mRNA expression levels. **(B)** In the prelimbic (PL) no significant differences was observed between groups for all examined genes. Values presented as relative quantification (RQ) to unexposed group and mean ± SEM per group. *significant difference between groups with *p* < 0.05.

Expression of *GAD65, GAD67, CCK* and *NPY* were assessed in the PL and IL sub-regions of the mPFC. In the IL, statistical analysis revealed that the controllable group exhibited lower expression levels of *GAD65, GAD67* and *CCK*, but no difference for *NPY* mRNA levels (*t*-test; *t*_(14)_ = 3.05, *p* < 0.01; *t*_(14)_ = 2.98, *p* < 0.05; *t*_(14)_ = 1.95, *p* < 0.05; *t*_(14)_ = 0.77, n.s; respectively. Figure 7A). In the PL no significant differences were observed between groups for all genes (*t*-test; *GAD65*: *t*_(14)_ = −0.68, n.s; *GAD67*: *t*_(14)_ = −0.34, n.s; *CCK*: *t*_(14)_ = −0.32, n.s; *NPY*: *t*_(14)_ = −1.36, n.s; Figure 7B).

## Discussion

The present study examined the effect of stress controllability on later fear extinction and alterations in GABAergic transmission in mPFC that may mediate this effect. Adult rats underwent prolonged controllable stress training, followed by extinction of either cued or contextual FC. Acquisition of long-term emotional controllability was verified by TWS re-exposure and EPM tests, in which resilient behavior was observed. In addition, controllable stress led to enhanced within-session extinction of cued, but not contextual FC. However, impaired extinction recall was detected in both extinction types following controllable stress. Moreover, exposure to controllable stress led to alterations in GABAergic marker expression in the IL but not in the PL.

Many studies examining controllable vs. uncontrollable experiences employed single day exposure protocols (Drugan et al., 1985; Heinsbroek et al., 1991; Tanaka, 1999; Brennan et al., 2003; Bland et al., 2006; Rozeske et al., 2009). A previous study conducted in our lab demonstrated that after a single day of exposure to controllable training, rats gained operational controllability (avoided the shock when presenting the cue). However, despite the avoidance response acquisition, rats still exhibited high levels of freezing to the context, indicating that they have not yet gained emotional controllability. Similar high freezing levels were observed in rats that were exposed to uncontrollable stress (Ilin and Richter-Levin, 2009). Thus, in the present study, in order to test the subsequent effects of not only operational but also emotional controllability, we employed the previously established 6 days controllable TWS training (Ilin and Richter-Levin, 2009). In the present study, performance improved along days of TWS training, and reached sufficient learning levels after the second day of training (indicated by reaching performance of more than 50% avoidance). It implies that animals gained operational controllability after 2 days of training. The exploration behavior during the TWS test 14 days after training validated that not only operational but also emotional controllability was acquired. Here, exploration levels of the controllable group were comparable to those of the unexposed group, implying reduced anxiety levels in the previously aversive training context. Two weeks after completion of TWS training, rats were also tested in the EPM in order to evaluate anxiety-related behavior. Whereas unexposed animals explored equally the open and closed arms, the controllable group tended to explore more the open arms (evident in several independent measures). Taken together, these results serve as a validation of a long lasting behavioral phenotype difference between the groups (both in and outside of the TWS context), induced by the training protocol. Moreover, they are in line with prior findings, of controllable stress’ beneficial effects (Lucas et al., 2014), implying the evolvement of resilience after exposure to a stressful and challenging background.

We next sought out to examine whether the beneficial emotional impact of controllable stress would be also expressed in subsequent extinction learning, despite the aversive experience component of the TWS training. The within-session decrease in freezing levels is a component by which extinction level can be evaluated. A steeper decrease in freezing levels, within each session, is considered to reflect better within-session extinction. Our results imply a differential impact of the initial TWS training on within-session extinction, dependent upon the FC paradigm type. Contextual fear within-session extinction of the controllable group was comparable to that of the unexposed group in each day. Distinctively, within-session of cued FC in the controllable group was facilitated, compared to unexposed animals. Overall, while previous experience to controllable stress had a beneficial impact on subsequent cued FC within-session extinction, it did not lead to such an advantage in contextual FC extinction.

The differential effect in within-session extinction may reflect a difference in the way each of the two extinction types correspond to the common prior learning experience in the TWS. It is possible that the extinction of cued FC is facilitated by the TWS training due to the resemblance in the learning processes. Extinction level is the behavioral outcome of two conflicting learning processes: the “excitatory” CS-US pairing trace acquired during the FC session, and the “inhibitory” CS-noUS trace which is attained during extinction (Eisenberg et al., 2003). Similarly, attainment of active avoidance requires two consecutive and opposing learning processes. The first and essential phase is conditioned reaction to the CS. Then, a suppression of this conditioned response is required in order to allow acquisition of instrumental avoidance contingency (Solomon and Wynne, 1954; Moscarello and LeDoux, 2013). Thus, gaining control over the US involves an inhibition of fear responses that can later lead to reduced anxiety in stressful situations (LeDoux, 2012). It is important to note that the facilitation of extinction we observed is probably not merely due to a sensory generalization process, because the two procedures are dependent upon different modalities. Alternatively, the observed facilitated within-session extinction of cued FC is suggested to stem from the prior experience, which involves a similar learning process in the TWS. In addition, the differential effect in within-session fear extinction cannot be explained by difference in FC acquisition level. In both FC paradigms, controllable and unexposed groups successfully acquired FC learning. This is in agreement with the lack of differences in PL gene expression, a region which is known to play a central role in FC acquisition (Corcoran and Quirk, 2007; Sierra-Mercado et al., 2011).

In addition to the controllable stress training impact on within-session fear extinction, another interesting effect was detected. In the extinction of both FC paradigms an impairment of long-term memory of the successful extinction of the previous day was observed in the controllable group, indicated by impaired between-session extinction recall. The controllable group displayed high levels of freezing in the beginning of each day in comparison to the low levels of fear memory that were established the day before. The fact that this phenomenon was manifested in the controllable group in both extinction paradigms, suggests that the prior TWS training experience served as a crucial factor leading to it. Importantly, impaired extinction recall is known to be a symptom in anxiety disorders and in animal models of stress (Graham and Milad, 2014).

In order to further understand the molecular background of controllability on our FC and extinction results we evaluated mRNA expression levels of GABAergic related markers in mPFC sub-regions. We performed an examination of interneuron-associated neuropeptides, due to their central role in neuronal activity modulation (Baraban and Tallent, 2004). We have previously demonstrated that the expression of GABAergic markers and neuropeptides is modulated in sub-regions of the hippocampus and the BLA after learning and emotional controllability (Hadad-Ophir et al., 2014). In this study, we extended the evaluation of GABAergic interneuron marker expression within mPFC sub regions, due to their well-known role in FC and extinction paradigms. The molecular results revealed alterations in GABAergic marker expression in the mPFC, in a sub-region dependent manner. While no changes in gene expression were observed in the PL, we detected significant alterations within the IL region. Expression of *GAD65, GAD67* and the neuropeptide *CCK* were reduced after controllable stress, while *NPY* expression remained unaffected. Interestingly, the combination of the behavioral and molecular findings echoes and complements previous findings by Izquierdo et al. (2006). In their work, brief uncontrollable stress led to morphological changes in IL, but not PL. In addition, the uncontrollable stress had no effect on cued FC acquisition; however, within-session extinction was attenuated, in comparison to unstressed controls. The neural dissociation was observed in the current study as well, and it similarly corresponded to the behavioral dissociation. This detected dissociation revealed a beneficial effect of controllable stress with regards to within-session extinction of cued fear, in contrast to the negative effect (Izquierdo et al., 2006) found.

The general trend of a reduced steady-state expression of markers for inhibitory transmission in the controllable group in comparison to the unexposed group may imply a shift in excitation-inhibition balance towards elevated transmission of the IL to downstream regions. One major target is the ITC that inhibits central amygdala neurons. In conjunction with the extinction facilitation that was observed in the current study, the results are in line with numerous studies that relate enhanced activation of IL neurons to reduced freezing during extinction training (e.g., Santini et al., 2004; Sierra-Mercado et al., 2011). However, elevation in IL transmission was also previously associated with intact or facilitated retrieval of extinction memory, a result that is seemingly contradictory to the current findings. In a recent study, Do-Monte et al. (2015) have challenged this view. The authors show that IL transmission is not necessary during the retrieval itself, but is crucial for the storage of extinction memory in target structures. It was suggested that intact IL activity during extinction leads to potentiation of BLA projections to the ITC, which mediates the reduction in freezing levels at the retrieval session. Thus, it is plausible that despite the proposed elevation in IL transmission following controllable stress, the plasticity in this downstream pathway is deficient in these animals, resulting in poor extinction retrieval. Further investigation of this issue will contribute to elucidate these effects.

In conclusion, it appears that controllable stress carries a protective effect on within-session extinction performance. However, it seems that prolonged controllable exposure does not completely abolish the harmful effects of the stressful experience, as controllable animals exhibit impaired fear extinction recall. We propose that stress controllability induces changes in the circuitry that controls extinction, and thereby is likely to underlie the observed facilitation of the within-session extinction. Resilience induced by controllable stress was previously proposed to evolve from a general resistance to later stressors, and was proposed to be a consequence of inhibitory control exerted by increased activity of the mPFC (Maier and Watkins, 2010). Our findings concur with this proposition, but also suggest a more refined prediction of controllable stress impact on coping with subsequent stressors. It suggests that the resilience induced by stress controllability does not lead to a generalized immunity against later aversive events, as previously proposed, but that the beneficial effect will be dependent upon the features of the controllable stress. Stressors that will resemble the initial experience will enable better coping as revealed by the dissociation between cued and contextual fear extinction following controllable stress training. The results of the current study serve as an example of a complex picture, in which prior stress sets the background for the outcome of subsequent stressful experiences. It demonstrates that the same experience may have a different impact, as a function of the environment in which the later experiences takes place, and the degree to which it shares common features with past learning. Moreover, the results suggest that a prior adverse experience, when controllable, can induce resilience in some aspects, as others remain impaired. Such complexity in considering the effects of stress on later coping is also suggested by the mismatch hypothesis (Schmidt, 2011). This point of view can be beneficial when trying to understand the considerable individual differences observed in anxiety-related pathologies, which may require more complex behavioral interpretations based on the personal history of stress.

## Author Contributions

OH-O, NB-E, and GR-L conceived and designed the experiments. OH-O and NB-E performed the experiments. OH-O, NB-E and GR-L analyzed and discussed the data. OH-O, NB-E and GR-L wrote the manuscript.

## Conflict of Interest Statement

The authors declare that the research was conducted in the absence of any commercial or financial relationships that could be construed as a potential conflict of interest.
